# Parotid gland masses: outcomes in the pediatric age group

**DOI:** 10.1186/s43046-023-00161-8

**Published:** 2023-02-06

**Authors:** Alaa Younes, Mohammad Fouad Taher, Iman Sidhom, Wael Zekri, Iman Zaky, Habiba Elfendy, Azza Niazy Taher, Suzan Adlan Khedr, Rana Gamal, Gehad Ahmed

**Affiliations:** 1 Department of Surgical Oncology, National Cancer Institute, Cairo University, Cairo, Egypt; 2 Department of Surgical Oncology, Children Cancer Hospital Egypt (CCHE, 57357), Cairo, Egypt; 3 Department of Pediatric Oncology, National Cancer Institute, Cairo University, Cairo, Egypt; 4 Department of Pediatric Oncology, Children Cancer Hospital Egypt (CCHE, 57357), Cairo, Egypt; 5 Department of Radiodiagnosis, National Cancer Institute, Cairo University, Cairo, Egypt; 6 Department of Radiodiagnosis, Children Cancer Hospital Egypt (CCHE, 57357), Cairo, Egypt; 7 Department of Pathology, National Cancer Institute, Cairo University, Cairo, Egypt; 8 Department of Pathology, Children Cancer Hospital Egypt (CCHE, 57357), Cairo, Egypt; 9 Department of Radiation Oncology, National Cancer Institute, Cairo University, Cairo, Egypt; 10 Department of Radiation Oncology, Children Cancer Hospital Egypt (CCHE, 57357), Cairo, Egypt; 11 Department of Anesthesia and Pain Management, National Cancer Institute, Cairo University, Cairo, Egypt; 12 Department of Clinical Research, Children Cancer Hospital Egypt (CCHE, 57357), Cairo, Egypt; 13 Department of General Surgery, Faculty of Medicine, Helwan University, Helwan, Egypt

**Keywords:** Pediatric parotid tumors, Parotidectomy, Parotid FNAC, Adjuvant radiotherapy, Neck dissection for parotid tumors

## Abstract

**Background:**

Childhood parotid neoplasms appear to have different characteristics from adults. This point, in addition to the rarity of these tumors, reflects the challenges faced in diagnosing and treating parotid neoplasms in children.

**Patients and methods:**

This retrospective study included all children who presented to the Children’s Cancer Hospital Egypt (CCHE, 57357) with parotid masses from January 2008 to December 2020.

**Results:**

Twenty-one patients were included. Malignant neoplasms were found in 12 (57.1%) of which mucoepidermoid carcinoma was the most common. Benign neoplasms were found in 6 (28.6%) all of them were pleomorphic adenoma, and non-neoplastic lesions were found in 3 (14.3%). Superficial, deep, or total parotidectomy was performed according to the involved lobes. The facial nerve was sacrificed in three cases because of frank invasion by the tumor. Neck dissection was considered in clinically positive lymph nodes and/or T3/4 masses. Complications occurred in 7 (33.3%) all were of the malignant cases. Adjuvant radiotherapy was restricted to high-risk cases (7 cases). Recurrence occurred in two cases, and one patient died of distant metastasis. Fine needle aspiration cytology (FNAC) showed 88.9% sensitivity and 100% specificity for diagnosing malignant neoplasms. The correlation of radiological and pathological staging was fair (66.74% for overall staging).

**Conclusions:**

Parotidectomy is the backbone treatment for benign and malignant pediatric parotid tumors. Neck nodal dissection should be considered after preoperative FNAC of suspicious nodes. Adjuvant radiotherapy is considered only in high-risk tumors. Preoperative FNAC of parotid masses and clinically suspicious lymph nodes is highly recommended.

## Introduction

Salivary gland tumors are rare in both adults and children [1, 2], representing less than 3% of head and neck tumors [3]. The most involved gland is the parotid [4]. Five percent of salivary gland tumors occur in patients under 18 years. Childhood parotid neoplasms appear to have different characteristics from those of adults. This point and the rarity of these tumors in pediatrics reflect the challenges faced in diagnosing and treating these tumors [4]. Among these differences are that pediatric parotid tumors are primarily malignant but often present at lower grades and stages than in adults with an overall better prognosis [3, 5]. In addition, and as long-term prognosis needs to be considered in children, particularly in low-/intermediate-grade tumors because of the long time to recurrence, the intensity and aggressiveness of treatment should be considered to differ between adults and children [5]. In adults, postoperative radiotherapy is generally performed for advanced cancer and high-grade malignancies, but indications for adjuvant radiation treatment remain unclear for pediatric cases [1].

Most studies report that parotidectomy is the backbone treatment for benign and malignant pediatric parotid tumors [4, 6]. Conservative (less than a lobectomy or enucleation) parotidectomy carries a great recurrence rate, particularly in pediatrics [4]. Simultaneous neck dissection is only recommended when clinically suspicious nodes are present because of the rarity of occult nodal metastasis documented in most studies [3, 4, 7]. There is no consensus on whether performing elective neck dissection or implementing the wait-and-see policy in cN0 cases is the best practice [8].

This work aimed to review and evaluate the management of pediatric parotid masses at the Children’s Cancer Hospital Egypt (CCHE, 57357) over 13 years. Surgical management, including the type and extent of resection and postoperative complications, the need for adjuvant treatment, the accuracy of diagnostic tools (radiological and cytopathological) in diagnosis and staging, and outcomes were evaluated.

## Patients and methods

Our retrospective case series study included all patients below 18 years who were diagnosed with a parotid mass and underwent surgical treatment at the CCHE from January 2008 to December 2020. We included any case with suspicious neoplasms due to a contradiction between FNAC and clinical, and radiological findings (thus we had 3 cases that finally proved to be non-neoplastic). We excluded those proved and confirmed by FNAC to be non-neoplastic, and supported by clinical and radiological findings, and referred them to other hospitals. Patients’ data were collected from medical records, and the following items were obtained and analyzed: age, gender, symptoms, preoperative imaging, fine needle aspiration cytology (FNAC) and/or preoperative biopsy results, histopathological examination of the surgical specimen, type and extent of surgery, postoperative complications, adjuvant treatment (radiotherapy or chemotherapy), follow-up (CT/MRI), and outcome (recurrence type, time, management, and survival). The clinical, radiological, and pathological staging was performed according to the 8th edition American Joint Committee on Cancer staging manual, considering the different pathological types and staging systems [9, 10].

Data were analyzed using IBM SPSS version 24 *(*Statistical Package for Social Sciences; SPSS Inc., Chicago, IL). Qualitative data were described as numbers and percentages. McNemar’s test was used to evaluate the concordance between categorical variables, and Cohen’s kappa was used to assess the interrater agreement for qualitative (categorical) items. The survival analysis was performed using the Kaplan–Meier method. A *p* value ≤ 0.05 was considered statistically significant; all tests were two-tailed. Overall survival (OS) was calculated from the day of diagnosis until the day of death or the latest follow-up. Disease-free survival (DFS) was calculated from the day of surgery until the day of recurrence, death, or the latest follow-up.

## Results

Twenty-one patients were diagnosed with parotid masses and underwent surgery. Clinicopathological features and a summary of the cases are provided in Table 1. The median age at presentation was 13 years (range 4–18); 57% of cases were more than 10 years old. Among the neoplastic cases, 57.1% were malignant (Table 2). Non-malignant cases presented as benign neoplastic (28.6%) and non-neoplastic (14.3%) (Table 3).

**Table 1:** Clinicopathological features of the patients

Character	No (*n* = 21)	%
Age (range 4–18 years)		
≤ 10 years	9	42.9
> 10 years	12	57.1
Sex		
Male	11	52.4
Female	10	47.6
Pathology		
Malignant	12	57.1
Benign	6 (all: pleomorphic adenoma)	28.6
Non-neoplastic	3	14.3
Surgery type		
Total parotidectomy	8	38.1
Superficial parotidectomy	12	57.1
Deep parotidectomy	1	4.8
Lymph node management		
Selective dissection	8	38.1
Radical neck dissection	2	9.5
None	11	52.4
Facial nerve management		
Preservation	18	85.7
Scarification	3	14.3
Surgical margin		
Positive	8	38.1
Negative	13	61.9
Clinicoradiological staging (*n* = 12)^a^		
I	1	8.3
II	1	8.3
III	6	50
IV	4	33.4
Pathological staging (*n* = 12)^a^		
I	1	8.3
II	6	50
III	2	16.7
IV	3	25
Recurrence ( local or distant)	2	9.5
Mortality	1	4.8
Recurrence in neoplastic cases (*n* = 18)	2	11
Mortality in malignant cases (*n* = 12)	1	8
Postoperative complications	7	33.3
Postoperative radiotherapy	11	52.4
Adjuvant	7	
Definitive	1	
Salvage	3	

^**a**^Benign neoplastic and non-neoplastic cases were not applicable for staging

**Table 2:** Summary of the 12 malignant cases

No.	Age (years)	Sex	FNAC	Other biopsy (incisional)	Surgery type	Surgical LN management	Pathology	Lymph nodes	Margins	RTH	Radiological TN/ stage	Pathological TN/ stage	Complications	Recurrence	Alive
1	8	F	No	Bx outside: mucoepidermoid carcinoma, low grade	Superficial parotidectomy	None	Mucoepidermoid carcinoma: low grade	0/1	Negative	No	T2N0/II	T2N0/II	No	No	Yes
2	16	F	Pleomorphic adenoma	No	Superficial parotidectomy	Selective	Mucoepidermoid carcinoma:low grade	1/5	Positive	Adjuvant	T3N0/III	T2N1/III	No	No	Yes
3	4	F	Mucoepidermoid carcinoma	No	Total parotidectomy	Selective	Mucoepidermoid carcinoma: intermediate grade	0/5	Positive	Adjuvant	T3N0/III	T2N0/II	Transient neuropraxia of facial nerve	No	Yes
4	5	M	Mucoepidermoid carcinoma	No	Superficial parotidectomy	Selective	Mucoepidermoid carcinoma: low grade	0/13	Negative	No	T3N0/III	T2N0/II	No	No	Yes
5	16	M	Mucoepidermoid carcinoma	No	Superficial parotidectomy	Selective	Mucoepidermoid carcinoma: intermediate grade	0/4	Negative	No	T3N0/III	T2N0/II	No	No	Yes
6	11	F	Malignant round and spindle cells with rhabdoid features	No	Superficial parotidectomy	None	Rhabdomyosarcoma: low grade	0/2	Positive	Definitive	T1N0/I	T1N0/I	Mandibular osteoradionecrosis	No	Yes
7	6	F	Myoepithelial carcinoma	No	Total parotidectomy	Selective	Epithelial myoepithelial carcinoma	0/7	Negative but close	Adjuvant	T3N0/III	T3N0/III	Transient neuropraxia of facial nerve	1 time	No
8	17	M	No	Bx outside; adenoid cystic carcinoma	Total parotidectomy	Selective	Adenoid cystic carcinoma	0/5	Negative	Adjuvant	T4N0/IV	T2N0/II	Facial palsy permenant (sacrificed)	No	Yes
9	13	M	No	Bx outside: nasopharyngeal carcinoma	Total parotidectomy	Radical dissection	Metastatic nasopharyngeal carcinoma	12/29	Positive	Definitive (salvage)	T4N1/IV	T4N1/IV	Facial palsy permanent (sacrificed)	No	Yes
10	18	F	Nasopharyngeal carcinoma	No	Total parotidectomy	Selective	Metastatic nasopharyngeal carcinoma	1/11	Positive	Definitive (salvage)	T4N0/IV	T4N1/IV	Mandibular osteoradionecrosis	No	Yes
11	18	M	Mucoepidermoid carcinoma	No	Total parotidectomy	Selective	Mucoepidermoid carcinoma: low grade	0/13	Positive	Adjuvant	T3N0/III	T2N0/II	No	No	Yes
12	18	M	Nasopharyngeal carcinoma	no	Total parotidectomy	Radical dissection	Metastatic nasopharyngeal carcinoma	10/28	Negative	Definitive (salvage)	T4N1/IV	T4N2/IV	Facial palsy permenant (sacrificed)	No	Yes

*F* Female, *M* Male, *FNAC* Fine needle aspiration cytology, *Bx* Incisional biopsy, *LN* Lymph nodes, *RTH* Radiotherapy

**Table 3:** Summary of the nine non-malignant cases

No.	Age (years)	Sex	FNAC	Other biopsy (incisional)	Surgery type	Pathology	Lymph nodes	Margins	RTH	Recurrence
1	18	F	Pleomorphic adenoma	No	Superficial parotidectomy	Pleomorphic adenoma	0/1	Negative	No	No
2	10	M	Pleomorphic adenoma	No	Superficial parotidectomy	Pleomorphic adenoma	0/1	Negative but close	No	No
3	16	M	Pleomorphic adenoma	No	Superficial parotidectomy	Pleomorphic adenoma	0/1	Negative with deficient capsule	No	3 times^a^
4	7	M	Atypical polymorphic lymphoid cells	No	Superficial parotidectomy	Chronic sialadenitis	0/3	Negative	No	No
5	7	M	Lymphoid cells	No	Superficial parotidectomy	Keratinous epidermoid cyst with chronic sialadenitis	0/1	Negative	No	No
6	5	M	Hamemorrhgic, vascular lesion	No	Superficial parotidectomy	Vascular malformation/hamartoma	0/1	Negative	No	No
7	13	F	No	Bx outside: pleomorphic adenoma	Deep parotidectomy	Pleomorphic adenoma	0/1	Negative	No	No
8	9	F	Pleomorphic adenoma	No	Superficial parotidectomy	Pleomorphic adenoma	0/3	Positive	Adjuvant	No
9	16	F	Pleomorphic adenoma	No	Total parotidectomy	Pleomorphic adenoma	0	Positive	Adjuvant	No

*F* Female, *M* Male, *FNAC* Fine needle aspiration cytology, *Bx* incisional biopsy, *LN* Lymph nodes, *RTH* Radiotherapy, *NA* Staging is not applicable, being non-malignant

^a^This patient underwent further excision up to completion to total parotidectomy with scarification of facial nerve after the third recurrence and received postoperative radiotherapy. The patient later underwent nerve grafting

No complications were documented in these cases, lymph nodes were not surgically approached, staging was not applicable, and all cases were alive at the end of the study

### Radiological staging

The concordance of radiological staging with pathological staging for malignant cases (*n* = 12) was moderate to fair at 50% for T staging, 83.3% for N staging, and 66.74% for overall staging.

### FNAC

FNAC was performed in 17 cases, and a biopsy was performed in four. Incisional biopsies were performed at the referring centers and revealed mucoepidermoid carcinoma (MEC), metastatic nasopharyngeal carcinoma (NPC), adenoid cystic carcinoma, and pleomorphic adenoma. Only 1 case out of the 17 FNAC was diagnosed incorrectly. Considering non-neoplastic and benign neoplastic conditions as one group (non-malignant) and malignant neoplasms as another group, FNAC had an 88.9% sensitivity and 100% specificity to malignant neoplasms. The positive predictive value was 100%, and the negative predictive value was 88.9%, with an accuracy of 94.1%.

### Surgical management

**Fig. 1:**
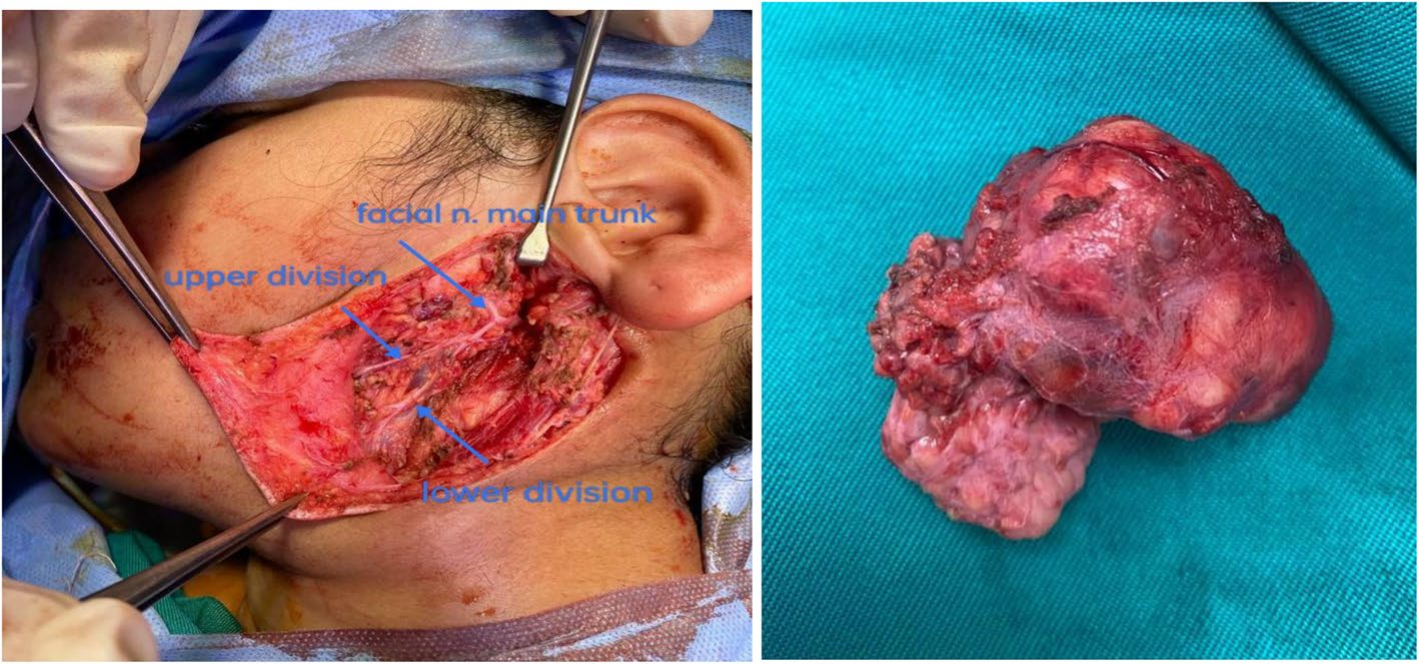
Pleomorphic adenoma: operative field, and specimen

**Fig. 2:**
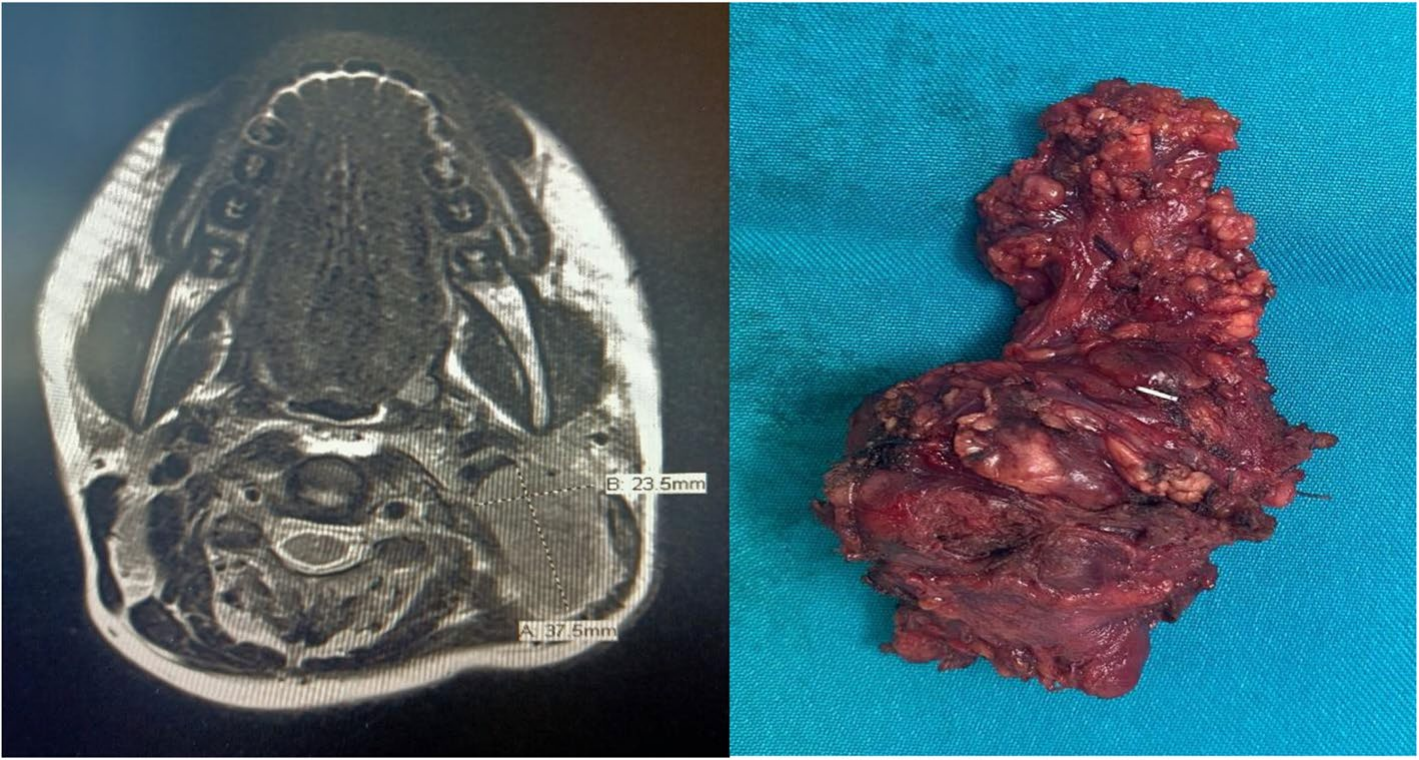
Mucoepidermoid carcinoma: MRI axial cut, and the specimen

Three types of parotidectomy surgery were performed based on the involved lobe pathology: superficial parotidectomy (12 cases), deep parotidectomy (1 case; pleomorphic adenoma), and total parotidectomy (8 cases). Figures 1 and 2  show some of our cases.

Approaches for lymph node management in malignant cases were adopted based on the clinical preoperative TN status. In cT3/4 N0 cases (*n* = 8), selective supraomohyoid dissection was performed. In cN+ cases (*n* = 2), radical neck dissection was performed by sacrificing the spinal accessory nerve in one case and sacrificing the spinal accessory and sternomastoid muscle in the other case, as these structures were intimately related to the involved nodes. In the other two malignant cases (cT1/T2 and N0) and the non-malignant cases, lymph nodes were not approached; nodes yielded in such cases were excised en bloc with the parotid mass.

Surgical and radiation therapy complications occurred only in malignant cases. The facial nerve was sacrificed in three patients because of frank invasion by the tumor. These patients later underwent staged reconstructive procedures. Two patients had transient neuropraxia of the facial nerve, which was managed and resolved with anti-inflammatory medications and physical therapy over 2 months. Postirradiation complications in the form of osteoradionecrosis of the mandible occurred in two cases, one of which required marginal mandibulectomy (these cases were NPCs with high dose of radiation received (6480 and 6600 cGy).

### Postoperative pathology

The histopathological examination of the surgical specimens revealed malignant neoplasms in 12 cases: 6 MECs, including four low-grade and 2 intermediate-grade, 3 metastatic NPCs, 1 adenoid cystic carcinoma, 1 epithelial myoepithelial carcinoma, and 1 rhabdomyosarcoma. Resection margins were involved in eight cases, two of which were pleomorphic adenomas, and received adjuvant radiation. Negative margins were obtained in the remaining 13 cases (Tables 2 and 3). Lymph nodes were pathologically positive in four patients (one low-grade MEC and three metastatic NPC cases). Among the non-malignant lesions, six were pleomorphic adenomas, and three were non-neoplastic lesions (one hamartoma and two chronic sialadenitis).

### Adjuvant therapy

Eleven patients received postoperative radiotherapy. Seven patients received postoperative radiotherapy as adjuvant treatment (three MECs with positive margins, two pleomorphic adenomas with positive margins, one epithelial myoepithelial carcinoma with close margins, and one adenoid cystic carcinoma). The three metastatic NPC cases received radiotherapy with salvage intent following surgery, and the rhabdomyosarcoma case received both chemotherapy and radiotherapy as definitive treatments.

### Recurrence, disease-free survival (DFS), and overall-survival (OS)

The median follow-up duration was 3.7 years (range 0.7–9 years). Two cases, one epithelial myoepithelial carcinoma, and one pleomorphic adenoma had recurrences after 13 and 23 months, respectively. The epithelial myoepithelial case developed local recurrence and nodal and distant lung metastases. The patient had permanent facial palsy, underwent tracheostomy due to extensive locoregional recurrence, received palliative care, and died of distant metastasis 3.5 years after surgery. The pleomorphic adenoma case had three local recurrences that were managed with repeated surgical excisions (a total parotidectomy was performed after the third recurrence with scarification of the facial nerve; nerve grafting was performed later for reconstruction). After the third recurrence, the patient received radiation therapy after resection.

**Fig. 3:**
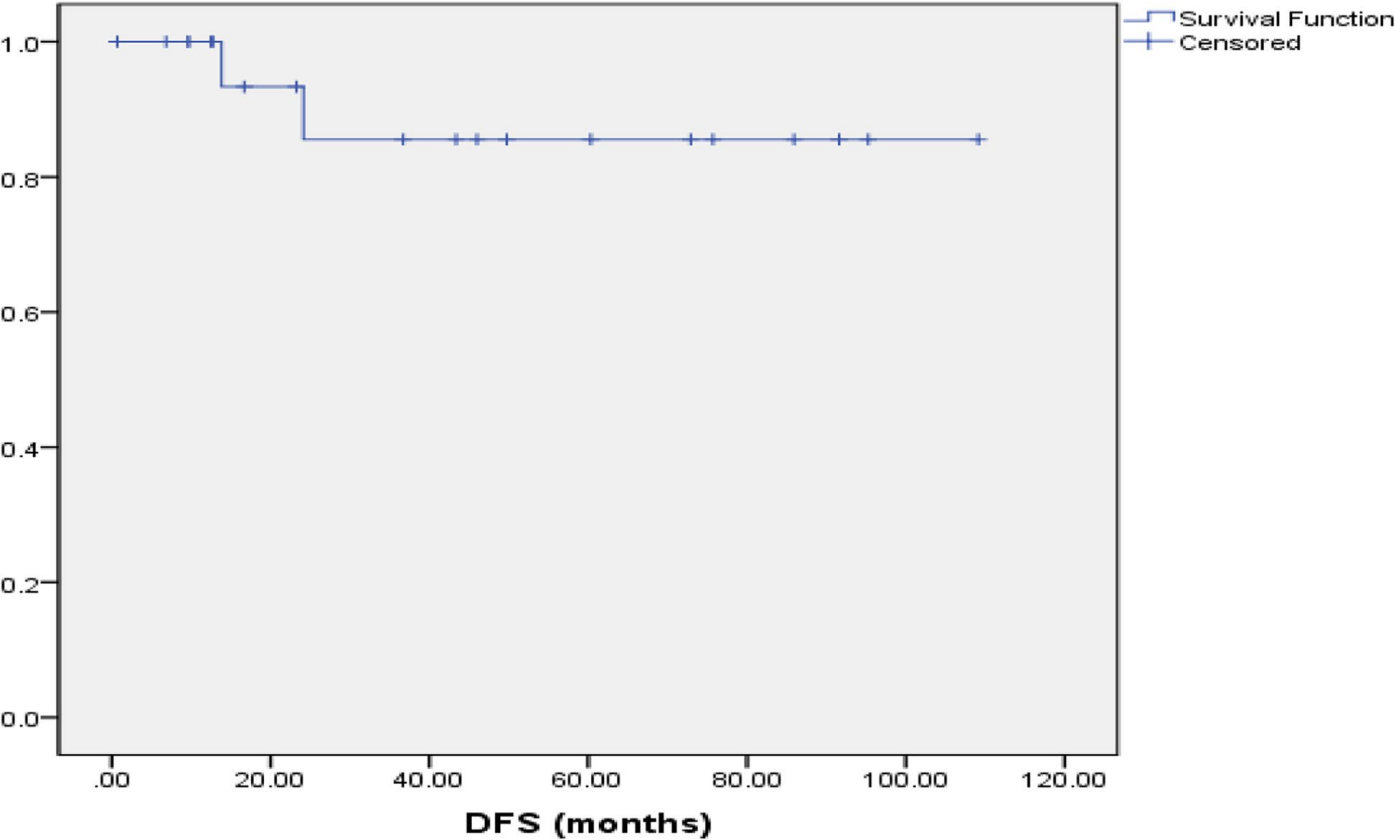
Disease-free survival (DFS)

**Fig. 4:**
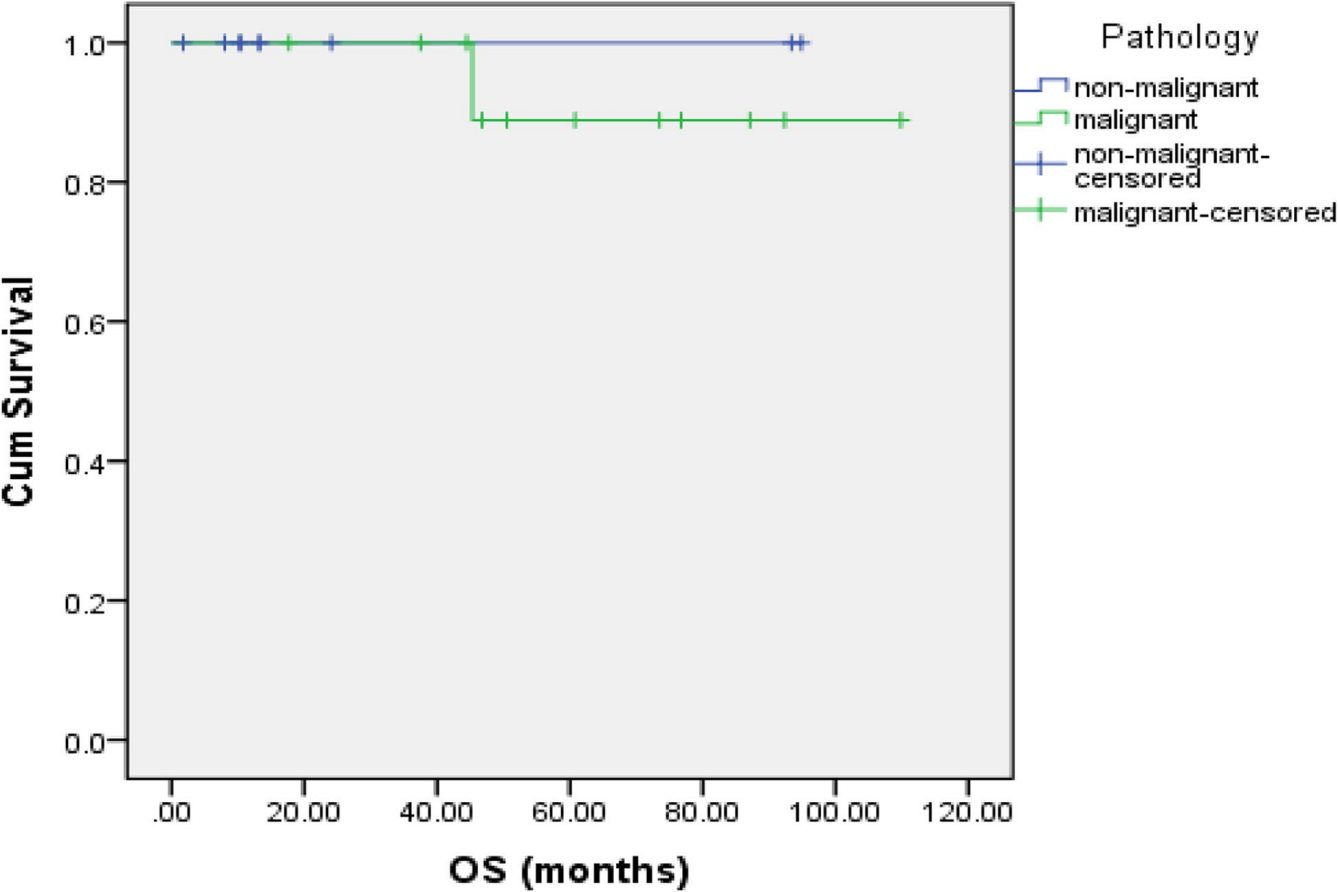
Overall survival (OS) by pathology

The recurrence rate was 11% and 8% for non-malignant and malignant cases, respectively. DFS was 93.3% ± 0.064 at 2 years and 85.6% ± 0.095 at the end of the study (Fig. 3). OS was 100% and 90% ± 0.1 at 5 years for non-malignant and malignant cases, respectively, and remained the same at 9 years (Fig. 4).

## Discussion

Both benign and malignant pediatric parotid tumors are rare [1, 2]. The most common benign parotid tumor is pleomorphic adenoma, and MEC is the most common malignant tumor in this age group [4]. The second-most frequent parotid malignant tumor is acinic cell carcinoma, followed by adenoid cystic carcinoma [2]. Our results were consistent with the typical presentation; all benign tumors were pleomorphic adenomas, and 66.7% of the primary malignant parotid tumors (*n* = 9) were MECs. Compared to adults, children are more likely to present with low-grade early-stage cancers [2]. Similarly, our study showed the predominance of early stages (I and II), which represented 77.8% of primary malignant cases and 58.3% of all malignant cases. After excluding metastatic malignant tumors, our study showed that 44.4% of the primary parotid malignant cases (four of nine) occurred in children less than 10 years old. This was contradictory to most reports in the literature. Bing et al. [3] reported a primary parotid malignant tumor in one of 13 cases (7.7%) less than 10 years old, and it was a lymphoma case in Ethunanada et al. [11] reported one out of three cases (33.33%) younger than 10 years. In another study, 28 of 284 patients (10%) less than 10 years old had primary parotid malignant tumors, compared with 101 (36%) from 10–15 years and 155 (54%) older than 15 years [12]. Lee et al. [4] found no malignant cases in patients less than 10 years old (Figs. 3 and 4).

In pleomorphic adenoma, the old debate between total versus superficial parotidectomy is now between parotidectomy (at least a lobectomy) and tumor-wide resection, including normal gland tissue (i.e., extracapsular resection) [13]. In our cases, the extent of the resection was dictated by the location of the lesion; four patients underwent superficial parotidectomy, one patient underwent deep lobe parotidectomy via a cervical incision for a deep lobe lesion, and another case underwent total parotidectomy. In all cases, the facial nerve was preserved. One case developed recurrence three times, as described above.

The relationship between the type of surgical treatment for MEC and survival has not been comprehensively examined. Ata-Ali et al. [14] recommended conservative (preserving facial nerve) total parotidectomy for all cases with MEC to decrease local relapse. However, the extent of parotidectomy in MEC is still debatable, and various approaches are still being considered. In the cases done in this study, superficial parotidectomy with facial nerve preservation was performed for superficial lobe lesions, and total parotidectomy was performed for deep lobe invasion by the tumor.

Sarcomas account for 1.5% of malignant tumors of the parotid gland. Rhabdomyosarcoma is the most common sarcoma arising from the parotid region during childhood and adolescence. Walterhouse et al. [15] reported 84% failure-free survival and 100% OS in parotid rhabdomyosarcoma cases. There was one rhabdomyosarcoma patient in our study who was treated by superficial parotidectomy and definitive chemotherapy and radiotherapy and was alive with no recurrence at the end of the follow-up period.

Epithelial myoepithelial carcinoma (EMC) is a rare malignant salivary gland tumor that accounts for 0.5% of all salivary gland malignancies. EMC has a high recurrence rate reaching 40%. Previous reports have shown a highly beneficial effect of adjuvant radiotherapy in enhancing local tumor control, especially for close margins and if the facial nerve is invaded [16]. We had only one case of EMC with a close margin, which had postoperative radiotherapy and developed locoregional and distant relapse after 13 months and transferred to palliative care.

NPC has a high incidence of nodal metastasis to cervical nodes, including retropharyngeal and level II nodes, followed by levels III, VA, and IV. However, parotid and supraclavicular lymph node groups have a very low risk for metastasis. The incidence of metastasis to parotid lymph nodes is less than 1%. The intraparotid lymphatics render the parotid uniquely vulnerable to tumor metastasis. So, it is crucial to have a careful clinical and radiographic evaluation of the parotid region of patients with NPC [17]. In our study, we had three cases of metastatic NPC to the parotid gland. These cases had their primary tumor treated earlier according to our hospital adopted protocols. Patients with early stage disease (stratum A) received radiation therapy only (61.2 Gy stage I, 66.6 Gy stage IIa), and patients with locoregionally advanced or metastatic disease (stratum B: IIb-V) received induction chemotherapy (3 cycles cisplatin and 5-FU) followed by consolidation chemoradiotherapy.

These NPC patients presented during their follow-up with recurrent neck and parotid masses, and were treated with salvage intent by surgical resection. Two underwent radical neck dissection (sacrificing the spinal accessory nerve and sternomastoid), and the facial nerve was invaded and sacrificed in both cases. The third case underwent selective supraomohyoid neck dissection.

The location of the primary tumor in the parotid gland influences the pattern of lymph node metastasis. The intraparotid lymph node is not the first echelon lymph node for regional spreading in all cases. Sometimes, lymph flow passes to the submandibular lymph nodes in lower pole lesions or into the accessory chain in posterior lesions [18]. Therefore, cases may have metastatic intraparotid lymph nodes with negative lateral neck nodes and vice versa [19]. Thus, the existence of metastatic intraparotid lymph nodes does not definitively signify lateral neck lymph node spread, which led to the proposal of a revised staging system dividing the regional lymph node staging system into parotid and cervical disease [20]. High-grade, tumor size of > 4 cm and age were reported as predictive factors for occult nodal metastasis [18].

Shinomiya et al. [21] found that the trend of lymph node metastasis varied between the cT2 and cT3/T4 patients. T2 cases had only intraparotid nodal spread, while T3/T4 cases had mostly lateral neck nodal spread. In cN+/pN+ patients, nodal metastasis passed to the cervical lymph nodes in 20% of cases (level I: 50%, level II: 70%, level III: 40%, level IV: 10%, and level V: 50%). In cN0 patients, occult nodal spread was restricted to levels I and II [21]. Therefore, modified radical neck dissection (levels I–V) was endorsed in patients with clinically positive lymph nodes, and elective cervical lymphadenectomy was strongly encouraged in patients with T3N0 or T4N0 disease (at least at levels I/II) [21]. Some authors also considered elective lymphadenectomy in high-grade tumors [22]. Pan et al. [23] evaluated the role of sentinel lymph node biopsy in cN0 and found a positive biopsy result in 33 of 198 (16.7%) patients and level II metastasis in 100% of patients. Still, the benefit of sentinel lymph node biopsy is not certainly clarified [8].

In our study, two cases of metastatic NPC with cN+ status underwent radical neck dissection (levels I–IV); their lymph nodes were pathologically positive. Selective supraomohyoid lymph node dissection (levels I–III) was performed in cases with cT3/T4 N0. One MEC case who underwent a selective dissection had a single positive pathological lymph node and had adjuvant radiotherapy due to lymph node involvement and a positive margin (whole neck and parotid). Of note, this patient had the only incorrect preoperative FNAC diagnosis in our study (as pleomorphic adenoma); selective dissection was performed based on the highly suspicious intraoperative circumstances despite the benign diagnosis by FNAC. In the other two malignant cases with cT2/T2 N0 and the non-malignant cases we did not approach the lymph nodes. The possibility of discordance between clinical and pathological node status led us to consider radiologically or clinically suspicious lymph nodes for FNAC before surgery.

In children, postoperative radiotherapy (PORT) as an adjuvant treatment should be cautiously considered. Radiotherapy complications can cause distorted facial growth, dental troubles, trismus, and osteoradionecrosis. There is also a higher risk of second malignancies in the exposed area [2]. Sultan et al. considered PORT in high-grade, advanced cancers with involved margins and lymph node spread. In our study, seven patients received adjuvant PORT due to high-risk features or pathological indications (positive or close margins, aggressive pathology) [24].

Preoperative cytology in parotid tumors is quite accurate and helpful in discriminating benign from malignant tumors and planning proper management [25]. To limit the risk of the misdiagnosis of pleomorphic adenoma, the Milan system classifies pleomorphic adenoma with non-classical aspects as salivary tumors of uncertain malignant potential. The final pathology may consist of a myoepithelial cell tumor, adenoid cystic carcinoma, carcinoma ex pleomorphic adenoma, or MEC [13].

Similar to our study, Ali et al. [4] reported 86% concordance of FNAC with histological results with a specificity and sensitivity of 98% and 84%, respectively, and diagnostic accuracy of 94%. In contrast, another study showed that FNAC had a diagnostic sensitivity of 100%, specificity of 25%, positive predictive value of 85%, negative predictive value of 100%, and accuracy of 85.7% for diagnosing benign parotid tumors. FNAC is indicated in all cases before surgery and is also considered for clinically or radiologically suspicious lymph nodes.

For benign tumors, low recurrence rates ranging from 0.5 to 5% after total parotidectomy for pleomorphic adenoma were reported [26, 27]. The median interval between the first operation and tumor recurrence was 3 to 15 years [26]. Sultan et al. [24] documented a 4% recurrence rate following parotidectomy after a median interval of 31 months. However, the recurrence rate was 50% if tumor enucleation was performed. Sultan et al. [24] and Fu et al. [26] both documented 100% survival in pleomorphic adenoma cases. Recurrences are difficult to treat, with an increased risk of facial nerve injury and development of other recurrences, hence PORT is to be considered judiciously when if satellite tumors are present, margins are positive or tumor spill is suspected [28]. It has been suggested that pleomorphic adenoma slowly takes on malignant features after repeated recurrences [29]. Malignant transformation is reported to occur in 5–40% of cases [30, 31]. Pelliccia et al. [27] found carcinoma in 16.1% of patients, two of which died after surgery of distant metastasis with or without local failure.

The recurrence rate is generally very low in malignant tumors. One study documented no recurrence over a long follow-up period [2]. Sultan et al. [24] documented a 25% occurrence of local relapses after parotidectomy in malignant tumors [24]. A 5-year DFS of 84.4% was documented by Feng et al. [6].

Allan et al. [12] reported 5-, 10-, and 20-year survival rates of 96%, 95%, and 83%, respectively. Other studies reported 5- and 10-year survival rates ranging from 81.1 to 100% and 66.7% to 94%, respectively [2, 24, 32, 33]. An overall 5-year survival of 100% was documented by Gontarz et al. [2], and Feng et al. [6] reported a 3-year OS of 100% and a 5-year OS of 95.8%. Feng et al. [6] also clarified that frequent recurrence in a short period was associated with a poor prognosis.

In our study, adverse events included two recurrences and one mortality as described in the results section.

## Conclusions

Parotidectomy with facial nerve preservation is the backbone treatment for benign and malignant pediatric parotid neoplasms. Neck nodal dissection is to be considered after preoperative FNAC of clinically suspicious nodes. Adjuvant radiotherapy is considered only in high-risk tumors because of the high rate of postirradiation complications in children. Preoperative FNAC of parotid masses and clinically suspicious lymph nodes is highly recommended.

## Limitations of the study

Many limitations faced our study. First, the retrospective nature of the study together with the very low number of cases hindered us to give solid conclusions. We, therefore, gave rather general recommendations.

## Data Availability

The datasets used/analyzed during this study are available from the corresponding author on request.

## Abbreviations

Bx: Biopsy
CCHE: Children’s Cancer Hospital Egypt
DFS: Disease free survival
EMC: Epithelial myoepithelial carcinoma
F: Female
FNAC: Fine needle aspiration cytology
LN: Lymph node
M: Male
MEC: Mucoepidermoid carcinoma
NA: Not applicable
NPC: Nasopharyngeal carcinoma
OS: Overall survival
PORT: Postoperative radiotherapy
RTH: Radiation therapy
